# Identification of a synonymous variant in TRIM59 gene for gastric cancer risk in a Chinese population

**DOI:** 10.18632/oncotarget.14075

**Published:** 2016-12-21

**Authors:** Dakui Luo, Younan Wang, Xiangkun Huan, Chi Huang, Chao Yang, Hao Fan, Zekuan Xu, Li Yang

**Affiliations:** ^1^ Department of General Surgery, The First Affiliated Hospital of Nanjing Medical University, Nanjing, Jiangsu, China; ^2^ Liver Transplantation Center of the First Affiliated Hospital and Key Laboratory on Living Donor Liver Transplantation, Ministry of Health, Nanjing Medical University, Nanjing, Jiangsu, China

**Keywords:** gastric cancer, TRIM59, synonymous variant, genotype, Chinese population

## Abstract

Tripartite motif 59 (TRIM59) is a novel oncogenic driver in gastric cancer (GC) that is implicated in disease progression as well as dismal survival. Genetic variants in peculiar gene are likely candidates for conferring hereditary susceptibility. The role of TRIM59 polymorphism in predicting the risk of malignant diseases and its relevance to TRIM59 expression have not been discussed. Using a HapMap tagSNPs approach, we screened three tag TRIM59 single nucleotide polymorphisms (SNPs) (rs1141023G>A, rs7629A>G, rs11706810T>C) which were genotyped in 602 GC patients and 868 healthy controls. Our study provided convincing result that carries of variant rs1141023A allele markedly increased GC risk (P=0.006). In comparison with the GG homozygotes, the variant GA heterozygotes demonstrated 1.50-fold elevated risk of GC (p=0.014, 95% confidence interval [CI] = 1.09–2.08). Subjects who carried the (GA+AA) genotypes of rs1141023 were associated with remarkable increased GC risk compared with the common genotype (P = 0.013, adjusted OR = 1.50, 95% CI = 1.09–2.05). Further stratified analyses displayed that the relationship between mutant genotype of rs1141023 and GC risk was more profound in male individuals. Intriguingly, there is no significant distinction of TRIM59 mRNA expression between rs1141023GA genotype and GG genotype in 44 normal gastric tissues. Taken together, our results suggest that rs1141023 polymorphism contributes to increased predisposition to GC and thus may be responsible for predicting early GC.

## INTRODUCTION

Gastric cancer (GC) represents the fifth commonly diagnosed carcinoma and the third prime cause of cancer-associated mortality on a world scale [1]. The inherit carcinogenesis of GC remains mainly unclear. Multiple genetic alterations as well as several etiologic factors are implicated in the development of GC [2]. The mysterious relationships between several particular polymorphisms and the risk of GC have been unveiled in previous epidemiology studies [3–6].

The tripartite motif (TRIM) family proteins are composed of a common N-terminal really interesting new gene (RING) finger domain and 1 or 2 B-boxes followed by coiled-coil sequences [7, 8]. The most of TRIM-containing proteins could be defined as E3 ubiquitin ligases for its RING-finger domain [9]. TRIM proteins were revealed to be relevant to diverse cancers via the regulation of transcriptional factors [10]. TRIM59, a novel biomarker in diverse carcinomas, is overexpressed in diverse tumors [11]. Zhou et al reported that TRIM59 promotes gastric tumor growth *in vitro* and *in vivo*. Its growth-enhancing function is initiated due to the ubiquitination and degradation of p53 [12]. However, the associations between hereditary polymorphisms of TRIM59 and the susceptibility to GC have not been discussed to date.

Single nucleotide polymorphisms (SNPs) are the most familiar form of DNA variation which can exert extensive influences on gene expression and a wide range of cellular functions [13–15]. Synonymous SNPs are previously regarded as invalid mutations, as they do not affect protein order [16, 17]. However, an increasing number of genomic data unveiled that synonymous mutations may have functional outcomes and could be targeted by natural selection [18, 19]. For instance, Bali V et al reported that synonymous codon change can alter drug sensitivity [20].

In the present study, we hypothesized that tagSNPs in TRIM59 gene may increase susceptibility to GC. To validate our speculation, we genotyped three TRIM59 tag SNPs (rs1141023, rs7629, rs11706810) in a retrospective case-control study with 602 patients who suffered from GC and 868 healthy volunteers in a Chinese Han population.

## RESULTS

### Demographic information

The distributions of demographic traits are detailed in Table 1. The present study included 602 GC patients and 868 age, gender matched cancer-free controls. There were no remarkable differences in the distributions of hypertension, diabetes mellitus and residence. However, there was larger proportion of smokers in the cases than in the controls.

**Table 1:** Demographic information

Characteristics	Cases (n = 602)	Controls (n = 868)	***P*** value
Age (y)*	60.6±10.7	59.5±12.8	0.058
Gender, (n (%))			
Female	164 (27.2)	273 (31.5)	
Male	438 (72.8)	595 (68.5)	0.083
Hypertension, (n (%))			
No	430 (71.4)	601 (69.2)	
Yes	172 (28.6)	267 (30.8)	0.367
Diabetes, (n (%))			
No	541 (89.9)	761 (87.7)	
Yes	61 (10.1)	107 (12.3)	0.193
Smoking, (n (%))			
Non-smokers	474 (78.7)	729 (84.0)	
Smokers	128 (21.3)	139 (16.0)	**0.010**
Residence, (n (%))			
Rural	358 (59.5)	473 (54.5)	
Urban	244 (40.5)	395 (45.5)	0.058
Tumor differentiation (n (%))			
Well	25 (4.2)		
Moderate	133 (22.1)		
Poor	444 (73.7)		
Depth of tumor infiltration (n (%))			
T1	90 (15.0)		
T2	67 (11.1)		
T3	265 (44.0)		
T4	180 (29.9)		
Lymph node metastasis (n (%))			
Negative	196 (32.6)		
Positive	406 (67.4)		
Localization (n (%))			
Cardia	265 (44.0)		
Noncardia	337 (56.0)		

*Median (25th-75th percentiles).

### Associations between TRIM59 alleles/genotypes and risk of gastric cancer

The allele as well as genotype frequencies of the selected SNPs and their relevance to risk of GC are summarized in Table 2 and Supplementary Table 1. The distributions of TRIM59 polymorphisms are in accord with Hardy-Weinberg equilibrium (p>0.05). Compared with the rs1141023 GG genotype, the variant GA, and GA+AA genotypes were statistically significantly implicated in increased risk of GC (p=0.014, adjusted OR=1.50, 95% CI=1.09-2.08 for GA; p=0.013, adjusted OR=1.50, 95% CI=1.09-2.05 for GA+AA) after modifying age, sex, smoking status, hypertension, diabetes mellitus and residence. The frequency of A allele was significantly increased among GC cases (p=0.006, OR=1.51, 95% CI=1.13-2.03). Three genetic models (additive, dominant and recessive) were applied for the analysis of the three SNPs. Logistic regression analyses showed that rs1141023 variant genotypes significantly increased gastric cancer risk in additive model and dominant model after adjustment for age, sex, smoking status, hypertension, diabetes mellitus and residence (additive model: P=0.013, adjusted OR=1.44, 95% CI=1.08-1.93; dominant model: P=0.013, adjusted OR=1.50, 95% CI=1.09-2.05) (Table 2). However, for the other two SNPs (rs7629 and rs11706810), no statistical differences were observed in our study (p>0.05, Supplementary Table 1).

**Table 2:** Association between TRIM59 gene polymorphisms and risk of gastric cancer (rs1141023)

genotype	Cases N (%)	Controls N (%)	Crude OR^a^ (95% CI^b^)	***P*** value	Adjusted OR (95% CI)*	***P*** value
overall	602	868				
GG	513 (85.2)	780 (89.9)	1		1	
GA	83 (13.8)	83 (9.6)	**1.52 (1.10-2.10)**	**0.011**	**1.50 (1.09-2.08)**	**0.014**
AA	6 (1.0)	5 (0.6)	1.83 (0.55-6.01)	0.323	1.76 (0.53-5.83)	0.356
Dominant model						
GG	513 (85.2)	780 (89.8)	1		1	
GA + AA	89 (14.8)	88 (10.2)	**1.54 (1.12-2.11)**	**0.007**	**1.50 (1.09-2.05)**	**0.013**
Recessive model						
GG + GA	596 (99.0)	863 (99.4)	1			
AA	6 (1.0)	5 (0.6)	1.74 (0.53-5.72)	0.363	1.68 (0.50-5.60)	0.398
Additive model			**1.48 (1.11-1.98)**	**0.008**	**1.44 (1.08-1.93)**	**0.013**
G	1109 (92.1)	1643 (94.6)	1			
A	95 (7.9)	93 (5.4)	**1.51 (1.13-2.03)**	**0.006**		
HWE^c^		0.093				

*Adjusted for age, sex, smoking status, residence, hypertension, and diabetes.

^a^OR, odds ratio.

^b^CI, confidence interval.

^c^HWE, Hardy–Weinberg expectations.

### Stratified analysis of the variants in TRIM59 gene and gastric cancer risk

Stratified by average age of controls (59 years), sex, smoking status and residence Subgroup analyses were to investigate the effects of mutant genotypes on the risk of GC. As demonstrated in Table 3, the variant genotypes (GA + AA) of rs1141023 dramatically contributed to the risk of GC in males (p=0.021, adjusted OR=1.56, 95% CI=1.07-2.28). Considering the rs7629 and rs11706810 polymorphisms, no significant associations between the two variants and age, sex, smoking status as well as residence were detected (Supplementary Table 2)

**Table 3:** Stratified analyses for TRIM59 genotypes in cases and controls (rs1141023)

Variable	n GA+AA (%)/n GG (%) for rs1141023		Allelic odds ratios and 95% confidence intervals for rs1141023	
	Cases	Controls	Adjusted OR (95% CI)*	***P*** value
Age (y), median				
≥59	58 (9.6)/315 (52.3)	39 (4.5)/383 (44.1)	1.46(0.97-2.21)	0.072
<59	31 (5.1)/198 (32.9)	49 (5.6)/397 (45.7)	1.48 (0.89-2.47)	0.129
Sex				
Females	21 (3.5)/143 (23.8)	28 (3.2)/245 (28.2)	1.35 (0.73-2.48)	0.341
Males	68 (11.3)/370 (61.5)	60 (6.9)/535 (61.6)	**1.56 (1.07-2.28)**	**0.021**
Smoking Status				
Smokers	21 (3.5)/107 (17.8)	11 (1.3)/128 (14.7)	2.10 (0.95-4.65)	0.068
Nonsmokers	68 (11.3)/406 (67.4)	77 (8.9)/652 (75.1)	1.39 (0.98-1.97)	0.068
Residence				
Rural	57 (9.5)/301 (50.0)	51 (5.9)/422 (48.6)	1.50 (0.99-2.26)	0.053
Urban	32 (5.3)/212 (35.2)	37 (4.3)/358 (41.2)	1.50 (0.90-2.48)	0.120

*Adjusted for age, sex, smoking status, residence, hypertension, and diabetes.

In our study, the associations between TRIM59 variant genotypes and clinicopathologic characteristics of gastric cancer patients were also evaluated (Table 4). The variant genotypes (GA + AA) of rs1141023 could increase the susceptibility to GC in subjects with advanced depth of tumor infiltration (T3 and T4), T3 (p=0.046, adjusted OR=2.37, 95% CI=1.01-5.55), T4 (p=0.033, adjusted OR=2.59, 95% CI=1.08-6.21). Besides that, positive correlation between the variant and those who suffered positive lymph node metastasis were observed (p=0.018, adjusted OR=1.93, 95% CI=1.12-3.31). No significant associations between the polymorphism of rs1141023 and tumor differentiation and location of the primary cancer respectively were found. In terms of the rs7629 and rs11706810 polymorphisms, no significant correlations were found between the variants and clinicopathologic variables (Supplementary Table 3).

**Table 4:** Associations between variant TRIM59 genotypes and clinicopathologic characteristics of gastric cancer (rs1141023)

Variable	GA+AA, GG for rs1141023		Allelic odds ratios and 95% confidence intervals for rs1141023	
	GA+AA, n	GG, n	Adjusted OR (95%CI)*	***P*** value
Tumor differentiation				
Well	2	23	1	
Moderate	19	114	1.55 (0.32-7.57)	0.586
Poor	68	376	1.99 (0.46-8.69)	0.361
Depth of tumor infiltration				
T1	7	83	1	
T2	8	59	1.56 (0.50-4.85)	0.445
T3	43	222	**2.37 (1.01-5.55)**	**0.046**
T4	31	149	**2.59 (1.08-6.21)**	**0.033**
Lymph node metastasis				
Negative	19	177	1	
Positive	70	336	**1.93 (1.12-3.31)**	**0.018**
Localization				
Cardia	41	224	1	
Noncardia	48	289	0.94 (0.60-1.49)	0.805

*Adjusted for age, sex, smoking status, residence, hypertension, and diabetes.

### Functional relevance of rs1141023 on TRIM59 expression level

**Figure 1:**
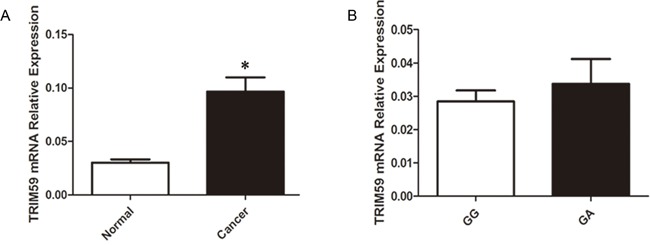
Relative expression levels of TRIM59 mRNA **A**. Expression level of TRIM59 mRNA in 44 paired gastric cancer tissues and normal gastric tissues: TRIM59 mRNA was significantly higher in gastric cancer tissues than normal gastric tissues; **B**. Relative expression level of TRIM59 mRNA grouped by rs1141023GG and rs1141023GA genotypes in 44 normal gastric tissues: there was no significant difference between GG and GA genotypes.

We wondered whether or not rs1141023 has an allele-specific influence on TRIM59 gene expression. The expression levels of TRIM59 mRNA were detected in forty-four pairs of cancerous and normal gastric tissues samples. Among the forty-four pairs of samples, thirty-four individuals carried the rs1141023 GG genotype, ten volunteers carried the GA genotype. Because of the limited number of AA genotype in general population, none of AA genotype was observed in above forty-four samples. As shown in Figure 1, TRIM59 mRNA level was highly overexpressed in cancerous tissues when compared with normal adjacent gastric tissues. There was no marked difference of TRIM59 mRNA between above two genotypes in normal gastric tissues.

## DISCUSSION

As far as we know, this study provided the initiative investigation of correlations between TRIM59 polymorphisms (rs1141023G>A, rs7629A>G, rs11706810T>C) and GC susceptibility in a Chinese Han nationality. Our study demonstrated that the synonymous variant rs1141023A (GA/AA) was related to a significantly increased risk of GC, especially in the male subjects. In the same time, the variant genotypes (GA + AA) of rs1141023 significantly increased the risk of GC in subjects with advanced depth of tumor. Intriguingly, TRIM59 mRNA level in subjects with rs1141023GG genotype was similar to those in subjects with rs1141023GA genotype in normal gastric tissues.

Increased risk of GC implicated in the variant genotypes of rs1141023 was more pronounced in males, which was shown in our study. Related outcome from the very early study had been revealed that the morbidity of gastric non-cardia cancer in males is two-fold than that in females. In addition, the ratio of cardia cancer is about 4.1:1 male to female in Chinese population [21]. These data demonstrated that the rs1141023 polymorphism may exert a preferable influence in males with GC.

The functions of the polymorphisms were evaluated followed stratification by clinicopathological characteristics of GC patients. We observed significant associations between rs1141023 polymorphism and patients whose tumor infiltration were T3 and T4 and those who suffered positive lymph node metastasis. Recent research revealed that TRIM59 expression was correlated positively with the pathologic grade of gastric cancer. Moreover, increased expression of TRIM59 was linked significantly to disadvantageous survival [12]. It is likely that the rs1141023 polymorphism may mediate higher expression level of TRIM59 protein and therefore play a role in the etiology of cancers with advanced tumor infiltration. We speculated that genetic variant may contribute to increase TRIM59 protein level, predicting poor clinical outcomes, which may partly attribute to positive lymph node metastasis. Further survival analysis between different genotypes is awaited.

The TRIM59 gene is a novel multiple carcinoma marker which may be used for molecular-targeted diagnosis and therapy of cancer. Besides that, TRIM59 gene as a proto-oncogene can affect both Ras and RB signal pathways just by transforming its function in DNA synthesis [11]. Zhou et al initially reported that when making a comparison with the adjacent normal gastric tissue, TRIM59 messenger RNA (mRNA) levels were increased markedly in gastric tumor samples. They confirmed that TRIM59 interacted physically with P53, accelerating its ubiquitination and degradation. Furthermore, TRIM59 repressed the expression of downstream molecules targeted by P53 [12]. In our previous study, we observed no overall association between the P53 Arg72Pro polymorphism and gastric cancer [22]. We assumed that Tag SNPs in the TRIM59 gene could influence differential binding affinities of P53, ultimately gain or loss the function of the downstream molecules. Hence, TRIM59 variant may increase gastric cancer risk.

Recognizing the precise mechanism of synonymous variant was the major challenge because the biology underlying the difference in susceptibility to developing GC could not be explained by a discrepancy in oncogenic properties. However, as more and more genomic data accumulated, many synonymous mutations no doubt have functional outcomes through affecting splicing events [23], messenger RNA stability, microRNA binding [24], and nucleosome formation [25]. Intriguingly, we observed that the synonymous variant rs1141023 was implicated in increased GC risk in Chinese Han nationality. We will explain the interested phenomenon in different levels. Duan et al revealed that synonymous mutations in the human dopamine receptor D2 decreased mRNA stability by altering mRNA secondary structure [26]. We hypothesized whether the synonymous region of SNP rs1141023 altered the TRIM59 secondary structure, stabilizing TRIM59 mRNA, eventually increasing its expression levels. However, variant genotype of the polymorphism showed no statistic difference in either gastric cancerous tissues or normal gastric tissues, leaving the mechanism in an inconsistent state. The general rule is that specific structure determines particular function. Promoter activity (gene expression), mRNA conformation (stability), and translational efficiency would be affected through SNPs which can occur between individuals within a population and are the simplest form of DNA variation [27]. Waldman showed that translation efficiency has been targeted by synonymous polymorphisms. Diverse codons have various efficiency of translation mainly due to the abundance of their respective transfer RNAs (tRNAs) in the ribosome [28]. Friedrich et al demonstrated the roles of two synonymous HTRA1 variants on protein structure and its interaction with TGF-β1 [29]. Although the rs1141023 polymorphism (G>A) did not affect TRIM59 mRNA expression levels, we speculated that rs1141023 polymorphism may be implicated in post-transcriptional process by targeting translation efficiency. Besides that, a previous study pointed out that the function of TRIM59 on gastric cancer was partly mediated by interacting physically with p53, increasing its ubiquitination and degradation [12]. We have reason to believe that alteration in TRIM59 structure may affect TRIM59 binding to p53. In particular conditions, synonymous polymorphisms could modify transporter specificity and protein stability, which is able to decipher differential phenotype [30]. Further precise mechanism underlying the function of the synonymous substitution remains to be unveiled. On the other hand, the relative limited sample size may account for the negative result partly. In prosperous study, larger tissue sample size is needed to validate TRIM59 mRNA expression among three different genotypes.

Several limitations should not be neglected. Above all, for the ethical reason, we did not detect helicobacter pylori for each subject. In addition, small sample size may lead to inadequate statistical power. The probability of type I error was should be minimized using all kinds of possible managements. We will keep recruiting the subjects in the future, appropriate sample size will make statistical power meet more rigorous standard in the subsequent studies. Beyond all that, selection bias should be addressed because all subjects were recruited from the identical hospital during the same period consecutively. Nevertheless, the genotype distribution among the controls in our research met the criterion of the Hardy–Weinberg expectations. Finally, one more issue remains to be solved, owing to our analysis restricted to Chinese Han population, studies on the basis of multi-populations are required to validate our findings.

In summary, we have provided initial evidence that rs1141023 residing in a synonymous region may affect the risk of GC. The variant genotypes (GA + AA) of rs1141023 significantly increased the risk of GC. Further precise functional studies validating our findings are awaited. All these indicated that TRIM59 and its genetic variations may be a promising biomarker for risk assessment, early diagnosis in GC.

## MATERIALS AND METHODS

### Ethics statement

This study was authorized by the ethics committee of the First Affiliated Hospital of Nanjing Medical University. Informed consents were gained from all recruited subjects.

### Study populations

The case-control study enrolled 602 GC cases and 868 cancer-free volunteers. Every patient was consecutively enlisted from the First Affiliated Hospital of Nanjing Medical University between May 2010 and October 2016 consecutively. The blood samples were derived from patients who were pathologically diagnosed of gastric cancer by endoscopic biopsy. The exclusion criteria for patients are composed of recurrent malignancies and those who accepted blood transfusion from other people; Subjects who underwent neoadjuvant chemotherapy or radiotherapy were also removed from this study. The cancer-free individuals were selected for matched age and sex with GC cases, and were randomly recruited from the uniform hospital during the same period. All the subjects were genetically irrelevant Han nationals and were from Jiangsu Province or its ambient districts. Information on age, gender, smoking history, residence, hypertension, diabetes mellitus were collected from medical records (for the GC patients) and questionnaire/interviews (for the healthy controls). The smokers were defined as individuals who smoked ≥10 cigarettes ceaselessly for more than 2 years. Hypertensive was deemed as persistent systolic blood pressure higher than 140 mmHg or a diastolic pressure greater than 90 mmHg. Patients receiving anti-hypertensive treatment also met the standard. Patients whose fasting plasma glucose ≥7 mmol/L or random plasma glucose ≥11.1 mmol/L were defined as diabetic mellitus. The residence was identified based on the medical records.

Gastric cancer tissues from a total of forty-four patients were collected from 2010 to 2014. The forty-four GC cases were confirmed by pathologic diagnosis and none of those had ever received neoadjuvant chemotherapy or radiotherapy.

### SNP selection

In terms of SNP selection, a HapMap tagSNPs (htSNPs) approach as the core criterion was used for analyzing the TRIM59 polymorphism. Tag SNPs were selected on the basis of genotype data for Han Chinese in Beijing from the HapMap database (HapMap Data Rel 27, Phase II+III, Feb09, on NCBI B36 assembly, dbSNP b126). The selection was conducted using the pairwise option in Haploview 4.2 software (Cambridge, MA, USA) and the threshold for analyses was set as r2 > 0.8 [31]. Functional SNPs such as those in the region of promoter, 3′ untranslated and exons are highly appreciated. Besides that, SNPs which implicated in other cancers demonstrated in previous studies will be also taken into consideration. Finally, on the basis of htSNPs approach, three tag SNPs (rs1141023G>A, rs7629A>G, rs11706810T>C) were screened and displayed.

### DNA extraction and genotyping

**Figure 2:**
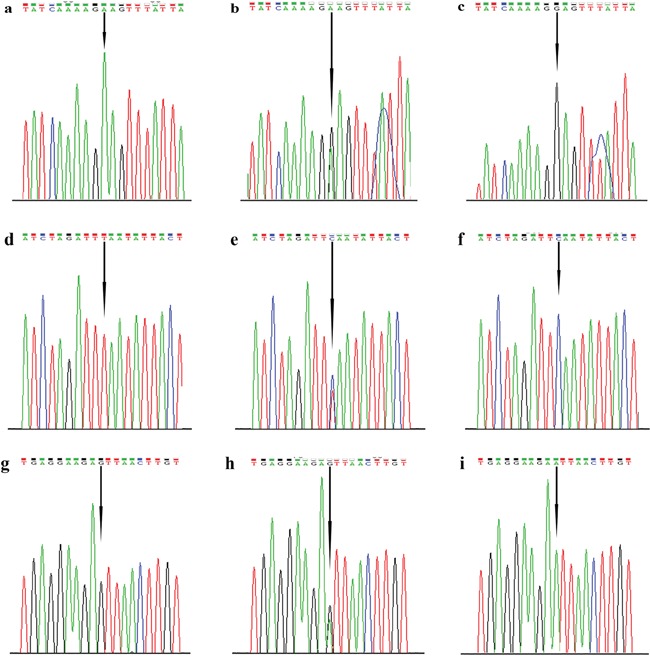
Direct sequencing results for tag SNPs in TRIM59 gene The polymorphisms were detected by TaqMan probe and confirmed by direct PCR sequencing. The single base indicated with a black arrowhead is the variant site. **a-c**. The AA, GA and GG genotypes of rs7629 by direct DNA sequencing, respectively. **d-f**. The TT, TC and CC genotypes of rs11706810 by direct DNA sequencing, respectively. **g-i**. The GG, GA and AA genotypes of rs1141023 by direct DNA sequencing, respectively.

Genomic DNA was derived from 2ml peripheral blood by using a blood DNA mini kit (Tiangen Biotech, Beijing China) according to the manufacturer's protocols. The purity and concentration of all samples were measured by NanoDrop Spectrophotometer (ND-1000) before the samples were diluted to a concentration of 10 ng/ul. Genotyping analysis of the three selected TRIM59 tagSNPs (rs1141023, rs7629, rs11706810) were performed using the TaqMan technology in 96-well plates. After that, the results were recognized with the Sequence Detection Software on a StepOnePlus instrument following the manufacturer's instructions (Applied Biosystems, Foster City, CA, USA). A polymerase chain reaction (PCR) was conducted in a 10μL reaction system which consists of 1×Taqman Universal PCR master mix, 500 nM of primer pairs, TaqMan minor groove binding probes and 10 ng of DNA template. The specific sequences of primers and probes for three tag SNPs are detailed in Table 5 (GENEray Biotechnology, Shanghai, China). Amplification was conducted under the following conditions: 50°C for 2 min followed by 95°C for 10 min and 60°C for 1 min for 40 cycles. About 10% of samples were randomly picked out for repeat assays, and the final concordance rate was 100%. The direct DNA sequencing technology was used to validate the genotypes of the three polymorphisms (Figure 2), and the results were consistent with previous genotypes exactly.

**Table 5:** The detailed sequences of primers and probes for tag SNPs

SNPs	Primer sequence (5′-3′)	Probe sequence
rs7629	F-TCAAAGAATTAGAAGTACCCAGAA	A: FAM-GACAGTGCAAATAAAATATCAAAAGAAGTTT-MGB
A>G	R- TGTATTTTTACCAGCAGGAAGATT	G: HEX-GACAGTGCAAATAAAATATCAAAAGGAGTTT-MGB
rs11706810	F- GCTGGCAAGACTTTACAACTTTACC	C: FAM-AGCAGTCGACGTACATTTAATCTAGATTCAAT-MGB
T>C	R- AGCATAGAATAGGGGTTTGGATAGA	T: HEX-AGCAGTCGACGTACATTTAATCTAGATTTAAT-MGB
rs1141023	F- AAATGACAAGCAGTATGGTAACAA	T: FAM-CATATGGGACAAGTTAATTCTTCCTC-MGB
G>A	R- CATGGCAGTACACGAGATCT	C: HEX-CATATGGGACAAGTTAACTCTTCCTC-MGB

### RNA isolation and reverse transcription real-time quantitative PCR analyses

Total RNA from frozen tissues were extracted with Trizol reagent (Invitrogen, Carlsbad, CA, USA) before every isolated RNA sample was converted to cDNA using Primescript RT Reagent (Takara, Otsu, Japan) following the directions. A reverse transcription real-time PCR (RT-PCR) was conducted employing StepOnePlus instrument (Applied Biosystems, Foster City, CA, USA) to quantify relative TRIM59 expression in these samples. The specific primers used for quantitative RT-PCR were detailed as follows: forward primer 5′-ATGATCCAAGGCGATAAGGAAGC-3′ and reverse primer 5′-ATCACAGAGAGCCGTTAGGAA-3′. The β-actin was selected as the endogenous control. The forward primer and reverse primer were listed as follows respectively: 5′-AGAAAATCTGGCACCACACC-3′ and 5′-TAGCACAGCCTGGATAGCAA-3′. A volume of 10 μl containing 5 μl Master mix, 0.2 μl primer and 100 ng cDNA was used for amplification reactions. The reaction condition was set at 95°C for 5m, followed by 40 cycles circulating 95°C for 10s and 60°C for 30s. We performed real-time PCR in StepOnePlus Real-Time PCR System (Applied Biosystems, Foster City, CA, USA) using SYBR Green Master Mix (Vazyme, Nanjing, China). All procedures were carried out in triplicate.

### Statistical analysis

SPSS22.0 software was selected for carrying out the statistical analysis in our experiment. P < 0.05 was deemed to meet the criterion of statistical significance. The whole tests were 2-sided. Distributions of demographic variables, allele and frequencies of genotype in cases as well as controls were calculated by the Pearson's χ2 test. Hardy-Weinberg equilibrium was figured out in controls using the goodness-of-χ2 test. Quantitative variables were recognized as medians and evaluated by the Mann-Whitney rank sum test. The odds ratios (OR) and 95% confidence intervals (CI) were calculated to evaluate the risk caused by a variant allele or genotype. Crude OR was evaluated using the Woolf approximation technique and the adjusted OR was computed by multivariate analysis of logistic regression, with adjustment for age, sex, hypertension, diabetes mellitus, smoking status, and residence. The relative expression levels of TRIM59 in all samples were calculated by the 2−Δct method compared with the levels of β-actin. The unpaired Student's t-test was applied to test statistically difference among the expression of TRIM59 with diverse rs1141023 genotypes. So is the expression of TRIM59 between gastric cancer tissues and normal gastric tissues. Statistical significance is presented as *P<0.05.

## SUPPLEMENTARY MATERIALS FIGURES AND TABLES


