# Supplementary material for: Identification of a synonymous variant in TRIM59 gene for gastric cancer risk in a Chinese population
Source: Oncotarget. 2016 Dec 21;8(7):11507–16. doi: 10.18632/oncotarget.14075 (PMC5355281; doi:10.18632/oncotarget.14075)
Supplement: Supplementary file 1 [file oncotarget-08-11507-s001.pdf]

Identification of a synonymous variant in TRIM59 gene for gastric cancer risk in a Chinese population

SUPPLEMENTARY TABLES

Supplementary Table 1: Association between TRIM59 gene polymorphisms and risk of gastric cancer (rs7629 and rs11706810)

genotype	Cases N (%)	Controls N (%)	Crude OR[a] (95% CI[b])	*P* value	Adjusted OR (95% CI)*	*P* value
overall	602	868				
rs7629						
AA	312 (51.8)	437 (50.3)	1		1	
AG	248 (41.2)	360 (41.5)	0.97 (0.78-1.20)	0.747	0.96 (0.77-1.19)	0.687
GG	42 (7.0)	71 (8.2)	0.83 (0.55-1.25)	0.367	0.83 (0.55-1.26)	0.386
Dominant model						
AA	312 (51.8)	437 (50.3)	1		1	
AG + GG	290 (48.2)	431 (49.7)	0.94 (0.77-1.16)	0.576	0.93 (0.76-1.15)	0.520
Recessive model						
AA + AG	560 (93.0)	797 (91.8)	1			
GG	42 (7.0)	71 (8.2)	0.84 (0.57-1.25)	0.395	0.84 (0.56-1.26)	0.400
Additive model			0.94 (0.79-1.10)	0.423	0.93 (0.79-1.10)	0.388
A	872 (72.4)	1234 (71.1)	1			
G	332 (27.6)	502 (28.9)	1.07 (0.91-1.26)	0.427		
HWE[c]		0.794				
rs1170680						
TT	382 (63.4)	532 (61.3)	1		1	
TC	187 (31.1)	283 (32.6)	0.92 (0.73-1.15)	0.472	0.90 (0.72-1.13)	0.372
CC	33 (5.5)	53 (6.1)	0.87 (0.55-1.37)	0.538	0.84 (0.53-1.34)	0.464
Dominant model						
TT	382 (63.4)	532 (61.3)	1		1	
TC + CC	220 (36.6)	236 (38.7)	0.91 (0.74-1.13)	0.400	0.89 (0.72-1.11)	0.293
Recessive model						
TT + TC	569 (94.5)	815 (93.9)	1			
CC	33 (5.5)	53 (6.1)	0.89 (0.57-1.40)	0.616	0.88 (0.56-1.38)	0.580
Additive model			0.93 (0.78-1.10)	0.383	0.91 (0.76-1.08)	0.289
T	951 (79.0)	1347 (77.6)	1			
C	253 (21.0)	389 (22.4)	1.09 (0.91-1.30)	0.368		
HWE[c]		0.066				

*Adjusted for age, sex, smoking status, residence, hypertension, and diabetes.

[a]OR, odds ratio.

[b]CI, confidence interval.

[c]HWE, Hardy–Weinberg expectations.

Supplementary Table 2: Stratified analyses for TRIM59 genotypes in cases and controls (rs7629 and rs11706810)

Variable	n GA+AA (%)/n GG (%) for rs7629		Allelic odds ratios and 95% confidence intervals for rs7629	
	Cases	Controls	Adjusted OR (95% CI)*	*P* value
Age (y), median				
≥59	193 (32.1)/180 (29.9)	202 (23.3)/220 (25.3)	1.03 (0.78-1.36)	0.847
<59	97 (16.1)/132 (21.9)	229 (26.4)/217 (25.0)	0.83 (0.60-1.15)	0.267
Sex				
Females	78 (13.0)/86 (14.3)	144 (16.6)/129 (14.9)	0.82 (0.56-1.22)	0.338
Males	212 (35.2)/226 (37.5)	287 (33.1)/308 (35.5)	0.99 (0.77-1.27)	0.913
Smoking Status				
Smokers	60 (10.0)/68 (11.3)	66 (7.6)/73 (8.4)	0.90 (0.54-1.50)	0.689
Nonsmokers	230 (38.2)/244 (40.5)	365 (42.1)/364 (41.9)	0.93 (0.74-1.18)	0.565
Residence				
Rural	171 (28.4)/187 (31.1)	249 (28.7)/224 (25.8)	0.82 (0.62-1.07)	0.168
Urban	119 (19.8)/125 (20.8)	182 (21.0)/213 (24.5)	1.15 (0.83-1.59)	0.397
**Variable**	**n GA+AA (%)/n GG (%) for rs11706810**		**Allelic odds ratios and 95% confidence intervals for rs11706810**	
Age (y), median				
≥59	143 (23.8)/230 (38.2)	161 (18.5)/261 (30.0)	0.92 (0.69-1.22)	0.562
<59	77 (12.8)/152 (25.2)	175 (20.2)/271 (31.2)	0.87 (0.61-1.23)	0.419
Sex				
Females	59 (9.8)/105 (17.4)	107 (12.3)/166 (19.1)	0.88 (0.58-1.32)	0.523
Males	161 (26.7)/277 (46.0)	229 (26.4)/366 (42.2)	0.91 (0.67-1.18)	0.462
Smoking Status				
Smokers	47 (7.8)/81 (13.5)	54 (6.2)/85 (9.8)	0.84 (0.49-1.42)	0.510
Nonsmokers	173 (28.7)/301 (50.0)	282 (32.5)/447 (51.5)	0.90 (0.71-1.14)	0.381
Residence				
Rural	136 (22.6)/222 (36.9)	206 (23.7)/267 (30.8)	0.78 (0.59-1.04)	0.090
Urban	84 (14.0/160 (26.6)	130 (15.0)/265 (30.5)	1.10 (0.78-1.55)	0.571

*Adjusted for age, sex, smoking status, residence, hypertension, and diabetes.

Supplementary Table 3: Associations between variant TRIM59 genotypes and clinicopathologic characteristics of gastric cancer (rs7629 and rs11706810)

Variable	AG+GG, AA for rs7629		Allelic odds ratios and 95% confidence intervals for rs7629	
	AG+GG, n	AA, n	Adjusted OR (95%CI)*	*P* value
Tumor differentiation				
Well	12	13	1	
Moderate	64	69	0.83 (0.33-2.05)	0.679
Poor	214	230	0.95 (0.42-2.15)	0.903
Depth of tumor infiltration				
T1	46	44	1	
T2	27	40	0.60 (0.30-1.19)	0.145
T3	115	150	0.75 (0.46-1.22)	0.250
T4	102	78	1.29 (0.77-2.17)	0.331
Lymph node metastasis				
Negative	92	104	1	
Positive	198	208	1.09 (0.77-1.54)	0.621
Localization				
Cardia	133	132	1	
Noncardia	157	180	0.88 (0.64-1.23)	0.457
**Variable**	**TC+CC, TT for rs11706810**		**Allelic odds ratios and 95% confidence intervals for rs11706810**	
	**TC+CC, n**	**TT, n**	**Adjusted OR (95%CI)***	***P* value**
Tumor differentiation				
Well	12	13	1	
Moderate	49	84	0.56 (0.23-1.39)	0.212
Poor	159	285	0.57 (0.25-1.29)	0.175
Depth of tumor infiltration				
T1	35	55	1	
T2	20	47	0.67 (0.33-1.36)	0.270
T3	89	176	0.82 (0.50-1.36)	0.445
T4	76	104	1.15 (0.68-1.95)	0.594
Lymph node metastasis				
Negative	71	125	1	
Positive	149	257	1.03 (0.72-1.47)	0.889
Localization				
Cardia	103	162	1	
Noncardia	117	220	0.86 (0.61-1.21)	0.387

*Adjusted for age, sex, smoking status, residence, hypertension, and diabetes.
